# Triazidotris[μ-2-(2-pyridyl)ethanolato]dicobalt(II) acetonitrile monosolvate

**DOI:** 10.1107/S1600536810046891

**Published:** 2010-11-20

**Authors:** Jie Yang, Shizheng Liu, Lei Lv, Dacheng Li

**Affiliations:** a School of Chemistry and Chemical Engineering, Liaocheng University, Shandong 252059, People’s Republic of China

## Abstract

In the title compound, [Co_2_(C_7_H_8_NO)_3_(N_3_)_3_]·CH_3_CN, the two Co^II^ ions in the dinuclear complex have different coordination environments, both in a distorted octa­hedral geometry. One Co^II^ atom is coordinated by three O atoms from the three 2-hy­droxy­ethyl­pyridine (HEP) bridging ligands, two N atoms from two HEP ligands and one azido ligand, while the other Co^II^ atom is coordinated by the same three O atoms, one N atom from an HEP ligand and two azido ligands. Weak inter­molecular C—H⋯N hydrogen bonds link the dinuclear complexes into corrugated layers parallel to the *bc* plane. These layers are further packed with the formation of channels propagating in [010] and filled with the disordered [in a ratio 0.691 (13):0.309 (13)] acetonitrile solvate mol­ecules.

## Related literature

For the crystal structures of cobalt complexes with related ligands, see: Lah *et al.* (2006 ▶); Cheng & Wei (2002 ▶). For general background to mol­ecules functioning as nanoscale magnets, see: Sanudo *et al.* (2003 ▶); Sessoli *et al.* (1993 ▶).
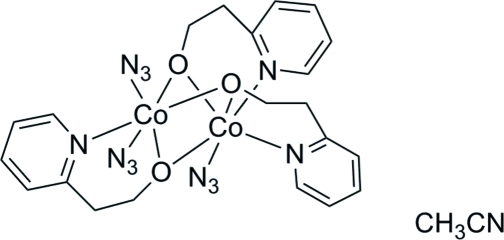


## Experimental

### 

#### Crystal data

[Co_2_(C_7_H_8_NO)_3_(N_3_)_3_]·C_2_H_3_N
*M* *_r_* = 651.44
Triclinic, 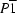

*a* = 10.8612 (11) Å
*b* = 10.9177 (12) Å
*c* = 13.3809 (14) Å
α = 90.299 (1)°
β = 112.659 (2)°
γ = 97.311 (1)°
*V* = 1449.8 (3) Å^3^
*Z* = 2
Mo *K*α radiation
μ = 1.19 mm^−1^
*T* = 298 K
0.28 × 0.23 × 0.11 mm

#### Data collection

Bruker SMART 1000 CCD diffractometer
Absorption correction: multi-scan (*SADABS*; Sheldrick, 1996) ▶ *T*_min_ = 0.731, *T*_max_ = 0.880
7657 measured reflections
5044 independent reflections
3337 reflections with *I* > 2σ(*I*)
*R*_int_ = 0.025

#### Refinement

*R*[*F* ^2^ > 2σ(*F* ^2^)] = 0.041
*wR*(*F* ^2^) = 0.098
*S* = 1.00
5044 reflections
399 parameters
H-atom parameters constrained
Δρ_max_ = 0.45 e Å^−3^
Δρ_min_ = −0.27 e Å^−3^

### 

Data collection: *APEX2* (Bruker, 2006 ▶); cell refinement: *SAINT* (Bruker, 2006 ▶); data reduction: *SAINT*; program(s) used to solve structure: *SHELXS97* (Sheldrick, 2008 ▶); program(s) used to refine structure: *SHELXL97* (Sheldrick, 2008 ▶); molecular graphics: *SHELXTL* (Sheldrick, 2008 ▶); software used to prepare material for publication: *SHELXTL*.

## Supplementary Material

Crystal structure: contains datablocks I, global. DOI: 10.1107/S1600536810046891/cv2795sup1.cif

Structure factors: contains datablocks I. DOI: 10.1107/S1600536810046891/cv2795Isup2.hkl

Additional supplementary materials:  crystallographic information; 3D view; checkCIF report

## Figures and Tables

**Table 1:** Hydrogen-bond geometry (Å, °)

*D*—H⋯*A*	*D*—H	H⋯*A*	*D*⋯*A*	*D*—H⋯*A*
C6A—H6A1⋯N2B^i^	0.97	2.42	3.382 (3)	169
C8A—H8A2⋯N1A^ii^	0.96	2.57	3.384 (4)	142

Symmetry codes: (i) 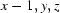; (ii) 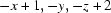.

**Fig. 1.**
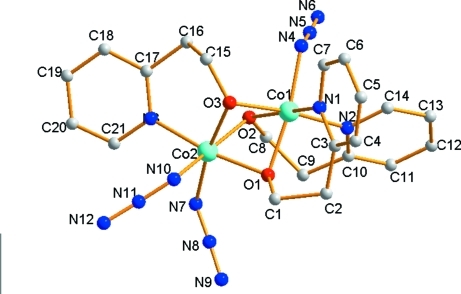
The molecular structure of (I) showing the atomic numbering and 30% probability displacement ellipsoids. The disordered solvent molecule and H atoms omitted for clarity.

## supplementary crystallographic information

### Comment

Many present and future specialized applications of magnets require monodisperse, nanoscale magnetic particles, and the discovery that individual molecules can function as nanoscale magnets was thus a significant development (Sanudo *et al.*, 2003; Sessoli *et al.*, 1993). We have synthesized the title compound, and characterized it by X-ray diffraction and elemental analysis which is reported in this paper.

In the title compound, (I) (Fig. 1), [Co_2_(C_7_H_9_NO)_3_(N_3_)_3_].CH_3_CN, two Co(II) ions in the dinuclear complex have different coordination environments both having distorted octahedral geometry. The bond lengths and angles in (I) are normal and correspond to those observed in related complexes (Lah *et al.*, 2006; Cheng *et al.*, 2002). Weak intermolecular C—H···N hydrogen bonds (Table 1) link the dinuclear complexes into corrugated layers parallel to *bc* plane. These layers are further packed with the formation of channels propagated in direction [010]  and filled with the disordered [in a ratio 0.691 (13):0.309 (13)] acetonitrile solvate molecules.

### Experimental

A mixtutre of solutions of CoCl_2_.6H_2_O(1 mmol, 238 mg) in methanol (10 ml) and acetonitrile (10 ml) was added Pyridine-2-ethanol(2 mmol, 246 mg) in 5 ml methanol, NaN_3_(2 mmol, 130 mg) and terramethylammonium hydroide(0.4 mmol, 165 mg, 25% solution in water), then stirred for 6 h. The resulting red solution was filtrated and was allowed to stand at room temperature for about three week, whereupon brown block crystal suitable for X-ray diffraction analysis was obtained.

### Refinement

All H atoms were placed in geometrically calculated positions, with C—H = 0.93–0.96 Å, and allowed to ride on their parent atoms, with *U*_iso_(H) = 1.2*U*_eq_(C) or 1.5*U*_eq_(C_methyl_).

### Crystal data

**Table:**

[Co_2_(C_7_H_8_NO)_3_(N_3_)_3_]·C_2_H_3_N	*Z* = 2
*M**_r_* = 651.44	*F*(000) = 668
Triclinic, *P*1	*D*_x_ = 1.492 Mg m^−^^3^
*a* = 10.8612 (11) Å	Mo *K*α radiation, λ = 0.71073 Å
*b* = 10.9177 (12) Å	Cell parameters from 2225 reflections
*c* = 13.3809 (14) Å	θ = 2.6–25.2°
α = 90.299 (1)°	µ = 1.19 mm^−^^1^
β = 112.659 (2)°	*T* = 298 K
γ = 97.311 (1)°	Block, brown
*V* = 1449.8 (3) Å^3^	0.28 × 0.23 × 0.11 mm

### Data collection

**Table:**

Bruker SMART 1000 CCD diffractometer	5044 independent reflections
Radiation source: fine-focus sealed tube	3337 reflections with *I* > 2σ(*I*)
graphite	*R*_int_ = 0.025
phi and ω scans	θ_max_ = 25.0°, θ_min_ = 1.7°
Absorption correction: multi-scan (*SADABS*; Sheldrick, 1996)	*h* = −12→12
*T*_min_ = 0.731, *T*_max_ = 0.880	*k* = −12→12
7657 measured reflections	*l* = −15→9

### Refinement

**Table:**

Refinement on *F*^2^	Primary atom site location: structure-invariant direct methods
Least-squares matrix: full	Secondary atom site location: difference Fourier map
*R*[*F*^2^ > 2σ(*F*^2^)] = 0.041	Hydrogen site location: inferred from neighbouring sites
*wR*(*F*^2^) = 0.098	H-atom parameters constrained
*S* = 1.00	*w* = 1/[σ^2^(*F*_o_^2^) + (0.0388*P*)^2^] where *P* = (*F*_o_^2^ + 2*F*_c_^2^)/3
5044 reflections	(Δ/σ)_max_ = 0.001
399 parameters	Δρ_max_ = 0.45 e Å^−^^3^
0 restraints	Δρ_min_ = −0.27 e Å^−^^3^

### Fractional atomic coordinates and isotropic or equivalent isotropic displacement parameters (Å^2^)

**Table:**

	*x*	*y*	*z*	*U*_iso_*/*U*_eq_	Occ. (<1)
Co1	0.79597 (5)	0.73623 (4)	0.18322 (4)	0.03535 (16)	
Co2	0.78387 (5)	0.97181 (5)	0.19775 (4)	0.03735 (16)	
N1	0.7998 (3)	0.6546 (3)	0.0525 (2)	0.0396 (8)	
N2	0.9332 (3)	0.6468 (3)	0.2842 (3)	0.0401 (8)	
N3	0.6333 (3)	1.0413 (3)	0.2150 (3)	0.0428 (8)	
N4	0.6574 (3)	0.6080 (3)	0.1861 (3)	0.0461 (9)	
N5	0.6549 (3)	0.5761 (3)	0.2696 (3)	0.0536 (10)	
N6	0.6448 (5)	0.5410 (5)	0.3463 (4)	0.1064 (19)	
N7	0.9149 (4)	1.0879 (3)	0.3095 (3)	0.0505 (9)	
N8	1.0220 (4)	1.1180 (3)	0.3044 (3)	0.0544 (10)	
N9	1.1266 (5)	1.1512 (4)	0.3049 (4)	0.0926 (16)	
N10	0.7804 (3)	1.0773 (3)	0.0821 (3)	0.0482 (9)	
N11	0.7882 (3)	1.1876 (3)	0.0911 (3)	0.0435 (8)	
N12	0.7913 (4)	1.2946 (3)	0.0908 (3)	0.0645 (11)	
N13	0.6698 (14)	0.2524 (18)	0.6694 (12)	0.215 (8)	0.691 (13)
N13'	0.438 (6)	0.163 (9)	0.532 (5)	0.22 (4)	0.309 (13)
O1	0.9186 (2)	0.8804 (2)	0.1873 (2)	0.0387 (6)	
O2	0.7787 (2)	0.8383 (2)	0.29203 (19)	0.0385 (6)	
O3	0.6765 (2)	0.8409 (2)	0.09277 (19)	0.0367 (6)	
C1	0.9670 (4)	0.8961 (4)	0.1030 (3)	0.0487 (11)	
H1A	1.0390	0.9653	0.1226	0.058*	
H1B	0.8949	0.9136	0.0367	0.058*	
C2	1.0189 (4)	0.7779 (4)	0.0853 (4)	0.0510 (11)	
H2A	1.0782	0.7957	0.0468	0.061*	
H2B	1.0707	0.7477	0.1550	0.061*	
C3	0.9046 (4)	0.6799 (4)	0.0213 (3)	0.0443 (10)	
C4	0.9015 (4)	0.6213 (4)	−0.0721 (4)	0.0590 (13)	
H4	0.9737	0.6394	−0.0931	0.071*	
C5	0.7932 (5)	0.5369 (4)	−0.1338 (4)	0.0652 (14)	
H5	0.7926	0.4961	−0.1952	0.078*	
C6	0.6867 (4)	0.5140 (4)	−0.1037 (3)	0.0537 (12)	
H6	0.6111	0.4589	−0.1452	0.064*	
C7	0.6926 (4)	0.5737 (3)	−0.0106 (3)	0.0459 (10)	
H7	0.6195	0.5576	0.0096	0.055*	
C8	0.8700 (4)	0.8420 (4)	0.4023 (3)	0.0478 (11)	
H8A	0.8360	0.7779	0.4387	0.057*	
H8B	0.8729	0.9211	0.4373	0.057*	
C9	1.0126 (4)	0.8245 (4)	0.4169 (3)	0.0496 (11)	
H9A	1.0436	0.8830	0.3744	0.059*	
H9B	1.0719	0.8422	0.4925	0.059*	
C10	1.0212 (4)	0.6958 (4)	0.3828 (3)	0.0436 (10)	
C11	1.1134 (4)	0.6259 (4)	0.4510 (4)	0.0595 (12)	
H11	1.1743	0.6605	0.5183	0.071*	
C12	1.1163 (5)	0.5073 (4)	0.4211 (4)	0.0647 (13)	
H12	1.1795	0.4613	0.4668	0.078*	
C13	1.0252 (5)	0.4571 (4)	0.3231 (4)	0.0585 (12)	
H13	1.0242	0.3758	0.3014	0.070*	
C14	0.9345 (4)	0.5285 (4)	0.2565 (3)	0.0483 (11)	
H14	0.8718	0.4936	0.1899	0.058*	
C15	0.5349 (3)	0.8193 (4)	0.0590 (3)	0.0429 (10)	
H15A	0.5028	0.7345	0.0298	0.052*	
H15B	0.4951	0.8730	0.0012	0.052*	
C16	0.4873 (4)	0.8415 (4)	0.1497 (3)	0.0475 (10)	
H16A	0.3919	0.8108	0.1247	0.057*	
H16B	0.5346	0.7947	0.2108	0.057*	
C17	0.5096 (4)	0.9748 (4)	0.1869 (3)	0.0476 (10)	
C18	0.4030 (5)	1.0300 (5)	0.1920 (4)	0.0644 (13)	
H18	0.3187	0.9833	0.1735	0.077*	
C19	0.4218 (5)	1.1530 (5)	0.2244 (4)	0.0764 (15)	
H19	0.3505	1.1907	0.2262	0.092*	
C20	0.5477 (5)	1.2189 (5)	0.2539 (4)	0.0714 (14)	
H20	0.5639	1.3018	0.2775	0.086*	
C21	0.6496 (5)	1.1608 (4)	0.2481 (3)	0.0531 (11)	
H21	0.7346	1.2066	0.2681	0.064*	
C22	0.577 (2)	0.270 (3)	0.600 (2)	0.221 (13)	0.691 (13)
C23	0.481 (3)	0.328 (3)	0.5030 (18)	0.287 (14)	0.691 (13)
H23A	0.4862	0.4141	0.5204	0.430*	0.691 (13)
H23B	0.3911	0.2874	0.4853	0.430*	0.691 (13)
H23C	0.5060	0.3184	0.4421	0.430*	0.691 (13)
C22'	0.414 (11)	0.059 (12)	0.509 (7)	0.27 (7)	0.309 (13)
C23'	0.394 (6)	−0.068 (7)	0.459 (5)	0.28 (5)	0.309 (13)
H23D	0.3369	−0.1224	0.4845	0.419*	0.309 (13)
H23E	0.4800	−0.0970	0.4800	0.419*	0.309 (13)
H23F	0.3532	−0.0665	0.3819	0.419*	0.309 (13)

### Atomic displacement parameters (Å^2^)

**Table:**

	*U*^11^	*U*^22^	*U*^33^	*U*^12^	*U*^13^	*U*^23^
Co1	0.0370 (3)	0.0337 (3)	0.0329 (3)	0.0039 (2)	0.0113 (3)	−0.0013 (2)
Co2	0.0417 (3)	0.0345 (3)	0.0354 (3)	0.0051 (2)	0.0146 (3)	−0.0005 (2)
N1	0.0397 (19)	0.0374 (19)	0.0384 (19)	0.0072 (15)	0.0111 (17)	−0.0022 (15)
N2	0.046 (2)	0.0364 (19)	0.0380 (19)	0.0086 (15)	0.0156 (17)	0.0027 (15)
N3	0.051 (2)	0.041 (2)	0.040 (2)	0.0118 (16)	0.0202 (18)	0.0026 (16)
N4	0.051 (2)	0.046 (2)	0.039 (2)	−0.0019 (17)	0.0179 (18)	0.0028 (17)
N5	0.051 (2)	0.053 (2)	0.049 (2)	−0.0040 (18)	0.014 (2)	0.008 (2)
N6	0.101 (4)	0.136 (4)	0.057 (3)	−0.034 (3)	0.019 (3)	0.023 (3)
N7	0.054 (2)	0.048 (2)	0.046 (2)	0.0016 (18)	0.018 (2)	−0.0076 (17)
N8	0.058 (3)	0.042 (2)	0.056 (2)	0.004 (2)	0.015 (2)	−0.0057 (18)
N9	0.065 (3)	0.089 (3)	0.120 (4)	−0.013 (3)	0.038 (3)	−0.019 (3)
N10	0.063 (2)	0.040 (2)	0.045 (2)	0.0055 (17)	0.0249 (19)	0.0029 (17)
N11	0.039 (2)	0.044 (2)	0.044 (2)	0.0024 (17)	0.0138 (17)	0.0017 (17)
N12	0.075 (3)	0.044 (2)	0.068 (3)	−0.002 (2)	0.023 (2)	0.006 (2)
N13	0.127 (11)	0.38 (2)	0.143 (12)	−0.007 (13)	0.069 (10)	0.085 (14)
N13'	0.13 (4)	0.43 (12)	0.12 (3)	0.03 (5)	0.07 (3)	0.03 (6)
O1	0.0394 (15)	0.0389 (15)	0.0370 (15)	0.0034 (12)	0.0148 (13)	−0.0042 (12)
O2	0.0452 (16)	0.0384 (15)	0.0325 (15)	0.0070 (12)	0.0154 (13)	0.0014 (12)
O3	0.0375 (15)	0.0366 (14)	0.0355 (14)	0.0041 (11)	0.0139 (13)	−0.0006 (12)
C1	0.051 (3)	0.049 (3)	0.049 (3)	−0.004 (2)	0.026 (2)	−0.003 (2)
C2	0.046 (2)	0.056 (3)	0.055 (3)	0.003 (2)	0.025 (2)	−0.007 (2)
C3	0.041 (2)	0.047 (3)	0.048 (3)	0.0094 (19)	0.019 (2)	−0.003 (2)
C4	0.057 (3)	0.068 (3)	0.057 (3)	0.000 (2)	0.031 (3)	−0.014 (3)
C5	0.070 (3)	0.072 (3)	0.053 (3)	0.002 (3)	0.025 (3)	−0.021 (3)
C6	0.053 (3)	0.054 (3)	0.047 (3)	−0.001 (2)	0.013 (2)	−0.012 (2)
C7	0.042 (2)	0.044 (2)	0.043 (2)	0.0028 (19)	0.008 (2)	−0.004 (2)
C8	0.063 (3)	0.047 (3)	0.030 (2)	0.008 (2)	0.014 (2)	−0.0031 (19)
C9	0.055 (3)	0.048 (3)	0.035 (2)	0.005 (2)	0.007 (2)	−0.004 (2)
C10	0.042 (2)	0.048 (3)	0.038 (2)	0.004 (2)	0.013 (2)	0.001 (2)
C11	0.059 (3)	0.065 (3)	0.045 (3)	0.014 (2)	0.009 (2)	0.003 (2)
C12	0.065 (3)	0.066 (3)	0.055 (3)	0.031 (3)	0.008 (3)	0.015 (3)
C13	0.069 (3)	0.047 (3)	0.063 (3)	0.022 (2)	0.025 (3)	0.007 (2)
C14	0.054 (3)	0.044 (3)	0.047 (3)	0.011 (2)	0.018 (2)	0.002 (2)
C15	0.035 (2)	0.046 (2)	0.042 (2)	0.0081 (18)	0.007 (2)	−0.0011 (19)
C16	0.039 (2)	0.051 (3)	0.051 (3)	0.007 (2)	0.016 (2)	0.004 (2)
C17	0.049 (3)	0.054 (3)	0.044 (3)	0.014 (2)	0.020 (2)	0.005 (2)
C18	0.055 (3)	0.070 (3)	0.074 (3)	0.016 (2)	0.029 (3)	−0.004 (3)
C19	0.068 (4)	0.082 (4)	0.087 (4)	0.032 (3)	0.033 (3)	−0.008 (3)
C20	0.086 (4)	0.060 (3)	0.074 (4)	0.025 (3)	0.033 (3)	−0.009 (3)
C21	0.065 (3)	0.047 (3)	0.052 (3)	0.014 (2)	0.025 (2)	−0.003 (2)
C22	0.126 (17)	0.43 (4)	0.123 (15)	0.03 (2)	0.067 (13)	0.03 (2)
C23	0.20 (2)	0.50 (5)	0.19 (2)	0.05 (3)	0.11 (2)	−0.03 (3)
C22'	0.18 (7)	0.4 (2)	0.16 (7)	0.04 (11)	0.03 (5)	0.04 (10)
C23'	0.21 (6)	0.42 (13)	0.16 (6)	0.01 (8)	0.03 (5)	0.14 (7)

### Geometric parameters (Å, °)

**Table:**

Co1—O3	1.907 (3)	C5—H5	0.9300
Co1—O2	1.909 (2)	C6—C7	1.378 (5)
Co1—O1	1.914 (2)	C6—H6	0.9300
Co1—N4	1.933 (3)	C7—H7	0.9300
Co1—N2	1.958 (3)	C8—C9	1.522 (5)
Co1—N1	1.977 (3)	C8—H8A	0.9700
Co1—Co2	2.6020 (7)	C8—H8B	0.9700
Co2—O3	1.916 (2)	C9—C10	1.500 (5)
Co2—O1	1.920 (2)	C9—H9A	0.9700
Co2—N10	1.926 (3)	C9—H9B	0.9700
Co2—N7	1.936 (3)	C10—C11	1.384 (6)
Co2—O2	1.942 (2)	C11—C12	1.362 (6)
Co2—N3	1.977 (3)	C11—H11	0.9300
N1—C7	1.349 (4)	C12—C13	1.361 (6)
N1—C3	1.353 (4)	C12—H12	0.9300
N2—C14	1.345 (5)	C13—C14	1.378 (6)
N2—C10	1.353 (5)	C13—H13	0.9300
N3—C21	1.343 (5)	C14—H14	0.9300
N3—C17	1.354 (5)	C15—C16	1.520 (5)
N4—N5	1.181 (5)	C15—H15A	0.9700
N5—N6	1.137 (5)	C15—H15B	0.9700
N7—N8	1.196 (5)	C16—C17	1.495 (5)
N8—N9	1.145 (5)	C16—H16A	0.9700
N10—N11	1.199 (4)	C16—H16B	0.9700
N11—N12	1.163 (4)	C17—C18	1.396 (5)
N13—C22	1.12 (2)	C18—C19	1.374 (6)
N13'—C22'	1.15 (12)	C18—H18	0.9300
O1—C1	1.419 (4)	C19—C20	1.369 (6)
O2—C8	1.423 (4)	C19—H19	0.9300
O3—C15	1.413 (4)	C20—C21	1.370 (6)
C1—C2	1.526 (5)	C20—H20	0.9300
C1—H1A	0.9700	C21—H21	0.9300
C1—H1B	0.9700	C22—C23	1.51 (3)
C2—C3	1.501 (5)	C23—H23A	0.9600
C2—H2A	0.9700	C23—H23B	0.9600
C2—H2B	0.9700	C23—H23C	0.9600
C3—C4	1.388 (5)	C22'—C23'	1.49 (9)
C4—C5	1.371 (6)	C23'—H23D	0.9600
C4—H4	0.9300	C23'—H23E	0.9600
C5—C6	1.360 (6)	C23'—H23F	0.9600
			
O3—Co1—O2	80.44 (10)	N1—C3—C4	120.1 (4)
O3—Co1—O1	78.49 (10)	N1—C3—C2	118.8 (3)
O2—Co1—O1	79.33 (10)	C4—C3—C2	121.0 (4)
O3—Co1—N4	96.18 (13)	C5—C4—C3	120.9 (4)
O2—Co1—N4	92.87 (12)	C5—C4—H4	119.6
O1—Co1—N4	171.13 (12)	C3—C4—H4	119.6
O3—Co1—N2	173.19 (11)	C6—C5—C4	118.8 (4)
O2—Co1—N2	95.74 (12)	C6—C5—H5	120.6
O1—Co1—N2	95.31 (12)	C4—C5—H5	120.6
N4—Co1—N2	89.60 (14)	C5—C6—C7	119.0 (4)
O3—Co1—N1	89.39 (12)	C5—C6—H6	120.5
O2—Co1—N1	169.41 (12)	C7—C6—H6	120.5
O1—Co1—N1	95.88 (12)	N1—C7—C6	122.9 (4)
N4—Co1—N1	91.11 (13)	N1—C7—H7	118.6
N2—Co1—N1	94.10 (13)	C6—C7—H7	118.6
O3—Co1—Co2	47.24 (7)	O2—C8—C9	113.9 (3)
O2—Co1—Co2	48.03 (7)	O2—C8—H8A	108.8
O1—Co1—Co2	47.35 (7)	C9—C8—H8A	108.8
N4—Co1—Co2	123.97 (10)	O2—C8—H8B	108.8
N2—Co1—Co2	126.20 (9)	C9—C8—H8B	108.8
N1—Co1—Co2	122.12 (9)	H8A—C8—H8B	107.7
O3—Co2—O1	78.13 (10)	C10—C9—C8	112.4 (3)
O3—Co2—N10	89.75 (12)	C10—C9—H9A	109.1
O1—Co2—N10	95.34 (13)	C8—C9—H9A	109.1
O3—Co2—N7	170.94 (13)	C10—C9—H9B	109.1
O1—Co2—N7	93.18 (13)	C8—C9—H9B	109.1
N10—Co2—N7	93.66 (15)	H9A—C9—H9B	107.9
O3—Co2—O2	79.39 (10)	N2—C10—C11	120.0 (4)
O1—Co2—O2	78.37 (10)	N2—C10—C9	118.5 (4)
N10—Co2—O2	168.33 (12)	C11—C10—C9	121.5 (4)
N7—Co2—O2	96.48 (13)	C12—C11—C10	121.0 (4)
O3—Co2—N3	96.99 (12)	C12—C11—H11	119.5
O1—Co2—N3	170.99 (12)	C10—C11—H11	119.5
N10—Co2—N3	92.19 (14)	C13—C12—C11	118.9 (4)
N7—Co2—N3	91.28 (14)	C13—C12—H12	120.6
O2—Co2—N3	93.36 (12)	C11—C12—H12	120.6
O3—Co2—Co1	46.96 (7)	C12—C13—C14	119.1 (4)
O1—Co2—Co1	47.16 (7)	C12—C13—H13	120.5
N10—Co2—Co1	121.82 (10)	C14—C13—H13	120.5
N7—Co2—Co1	124.69 (11)	N2—C14—C13	122.4 (4)
O2—Co2—Co1	46.96 (7)	N2—C14—H14	118.8
N3—Co2—Co1	124.18 (10)	C13—C14—H14	118.8
C7—N1—C3	118.3 (3)	O3—C15—C16	113.4 (3)
C7—N1—Co1	119.3 (3)	O3—C15—H15A	108.9
C3—N1—Co1	122.4 (2)	C16—C15—H15A	108.9
C14—N2—C10	118.6 (4)	O3—C15—H15B	108.9
C14—N2—Co1	118.1 (3)	C16—C15—H15B	108.9
C10—N2—Co1	123.2 (3)	H15A—C15—H15B	107.7
C21—N3—C17	118.0 (3)	C17—C16—C15	113.2 (3)
C21—N3—Co2	119.7 (3)	C17—C16—H16A	108.9
C17—N3—Co2	122.1 (3)	C15—C16—H16A	108.9
N5—N4—Co1	120.4 (3)	C17—C16—H16B	108.9
N6—N5—N4	175.6 (5)	C15—C16—H16B	108.9
N8—N7—Co2	118.0 (3)	H16A—C16—H16B	107.7
N9—N8—N7	176.0 (5)	N3—C17—C18	120.4 (4)
N11—N10—Co2	122.8 (3)	N3—C17—C16	119.7 (3)
N12—N11—N10	174.5 (4)	C18—C17—C16	119.9 (4)
C1—O1—Co1	119.9 (2)	C19—C18—C17	120.4 (4)
C1—O1—Co2	122.1 (2)	C19—C18—H18	119.8
Co1—O1—Co2	85.49 (10)	C17—C18—H18	119.8
C8—O2—Co1	121.3 (2)	C20—C19—C18	118.6 (4)
C8—O2—Co2	122.7 (2)	C20—C19—H19	120.7
Co1—O2—Co2	85.01 (10)	C18—C19—H19	120.7
C15—O3—Co1	124.3 (2)	C19—C20—C21	119.0 (4)
C15—O3—Co2	121.4 (2)	C19—C20—H20	120.5
Co1—O3—Co2	85.80 (10)	C21—C20—H20	120.5
O1—C1—C2	108.9 (3)	N3—C21—C20	123.6 (4)
O1—C1—H1A	109.9	N3—C21—H21	118.2
C2—C1—H1A	109.9	C20—C21—H21	118.2
O1—C1—H1B	109.9	N13—C22—C23	162 (3)
C2—C1—H1B	109.9	N13'—C22'—C23'	167 (10)
H1A—C1—H1B	108.3	C22'—C23'—H23D	109.5
C3—C2—C1	111.0 (3)	C22'—C23'—H23E	109.5
C3—C2—H2A	109.4	H23D—C23'—H23E	109.5
C1—C2—H2A	109.4	C22'—C23'—H23F	109.4
C3—C2—H2B	109.4	H23D—C23'—H23F	109.5
C1—C2—H2B	109.4	H23E—C23'—H23F	109.5
H2A—C2—H2B	108.0		
			
O2—Co1—Co2—O3	−121.83 (14)	N10—Co2—O1—C1	−6.8 (3)
O1—Co1—Co2—O3	118.84 (14)	N7—Co2—O1—C1	−100.7 (3)
N4—Co1—Co2—O3	−63.50 (16)	O2—Co2—O1—C1	163.3 (3)
N2—Co1—Co2—O3	177.63 (15)	N3—Co2—O1—C1	139.7 (7)
N1—Co1—Co2—O3	53.28 (14)	Co1—Co2—O1—C1	122.7 (3)
O3—Co1—Co2—O1	−118.84 (14)	O3—Co2—O1—Co1	−40.86 (10)
O2—Co1—Co2—O1	119.33 (14)	N10—Co2—O1—Co1	−129.47 (12)
N4—Co1—Co2—O1	177.66 (16)	N7—Co2—O1—Co1	136.55 (13)
N2—Co1—Co2—O1	58.79 (15)	O2—Co2—O1—Co1	40.58 (9)
N1—Co1—Co2—O1	−65.56 (15)	N3—Co2—O1—Co1	17.0 (8)
O3—Co1—Co2—N10	−54.07 (15)	O3—Co1—O2—C8	−164.8 (3)
O2—Co1—Co2—N10	−175.90 (16)	O1—Co1—O2—C8	−84.8 (3)
O1—Co1—Co2—N10	64.77 (16)	N4—Co1—O2—C8	99.4 (3)
N4—Co1—Co2—N10	−117.57 (17)	N2—Co1—O2—C8	9.5 (3)
N2—Co1—Co2—N10	123.56 (16)	N1—Co1—O2—C8	−148.7 (6)
N1—Co1—Co2—N10	−0.79 (16)	Co2—Co1—O2—C8	−125.6 (3)
O3—Co1—Co2—N7	−175.47 (16)	O3—Co1—O2—Co2	−39.24 (9)
O2—Co1—Co2—N7	62.70 (16)	O1—Co1—O2—Co2	40.73 (10)
O1—Co1—Co2—N7	−56.63 (17)	N4—Co1—O2—Co2	−135.03 (12)
N4—Co1—Co2—N7	121.03 (18)	N2—Co1—O2—Co2	135.08 (11)
N2—Co1—Co2—N7	2.16 (17)	N1—Co1—O2—Co2	−23.1 (7)
N1—Co1—Co2—N7	−122.19 (17)	O3—Co2—O2—C8	163.5 (3)
O3—Co1—Co2—O2	121.83 (14)	O1—Co2—O2—C8	83.6 (3)
O1—Co1—Co2—O2	−119.33 (14)	N10—Co2—O2—C8	141.8 (6)
N4—Co1—Co2—O2	58.33 (16)	N7—Co2—O2—C8	−8.4 (3)
N2—Co1—Co2—O2	−60.54 (15)	N3—Co2—O2—C8	−100.0 (3)
N1—Co1—Co2—O2	175.11 (14)	Co1—Co2—O2—C8	124.3 (3)
O3—Co1—Co2—N3	64.34 (15)	O3—Co2—O2—Co1	39.18 (9)
O2—Co1—Co2—N3	−57.50 (15)	O1—Co2—O2—Co1	−40.74 (9)
O1—Co1—Co2—N3	−176.82 (15)	N10—Co2—O2—Co1	17.5 (7)
N4—Co1—Co2—N3	0.84 (17)	N7—Co2—O2—Co1	−132.67 (12)
N2—Co1—Co2—N3	−118.03 (16)	N3—Co2—O2—Co1	135.66 (12)
N1—Co1—Co2—N3	117.62 (16)	O2—Co1—O3—C15	−85.8 (2)
O3—Co1—N1—C7	−75.3 (3)	O1—Co1—O3—C15	−166.7 (3)
O2—Co1—N1—C7	−91.2 (7)	N4—Co1—O3—C15	6.1 (3)
O1—Co1—N1—C7	−153.7 (3)	N2—Co1—O3—C15	−142.0 (9)
N4—Co1—N1—C7	20.9 (3)	N1—Co1—O3—C15	97.2 (3)
N2—Co1—N1—C7	110.5 (3)	Co2—Co1—O3—C15	−125.6 (3)
Co2—Co1—N1—C7	−111.4 (3)	O2—Co1—O3—Co2	39.83 (9)
O3—Co1—N1—C3	102.3 (3)	O1—Co1—O3—Co2	−41.11 (9)
O2—Co1—N1—C3	86.4 (7)	N4—Co1—O3—Co2	131.71 (12)
O1—Co1—N1—C3	23.9 (3)	N2—Co1—O3—Co2	−16.4 (10)
N4—Co1—N1—C3	−161.5 (3)	N1—Co1—O3—Co2	−137.24 (11)
N2—Co1—N1—C3	−71.9 (3)	O1—Co2—O3—C15	169.1 (3)
Co2—Co1—N1—C3	66.2 (3)	N10—Co2—O3—C15	−95.4 (3)
O3—Co1—N2—C14	−156.3 (9)	N7—Co2—O3—C15	152.4 (8)
O2—Co1—N2—C14	148.3 (3)	O2—Co2—O3—C15	88.9 (3)
O1—Co1—N2—C14	−132.0 (3)	N3—Co2—O3—C15	−3.2 (3)
N4—Co1—N2—C14	55.4 (3)	Co1—Co2—O3—C15	128.1 (3)
N1—Co1—N2—C14	−35.7 (3)	O1—Co2—O3—Co1	41.02 (10)
Co2—Co1—N2—C14	−171.2 (2)	N10—Co2—O3—Co1	136.53 (12)
O3—Co1—N2—C10	27.7 (11)	N7—Co2—O3—Co1	24.4 (8)
O2—Co1—N2—C10	−27.7 (3)	O2—Co2—O3—Co1	−39.18 (9)
O1—Co1—N2—C10	52.0 (3)	N3—Co2—O3—Co1	−131.30 (11)
N4—Co1—N2—C10	−120.6 (3)	Co1—O1—C1—C2	−49.8 (4)
N1—Co1—N2—C10	148.3 (3)	Co2—O1—C1—C2	−154.5 (2)
Co2—Co1—N2—C10	12.8 (3)	O1—C1—C2—C3	78.0 (4)
O3—Co2—N3—C21	−154.7 (3)	C7—N1—C3—C4	−1.5 (6)
O1—Co2—N3—C21	148.7 (7)	Co1—N1—C3—C4	−179.1 (3)
N10—Co2—N3—C21	−64.7 (3)	C7—N1—C3—C2	174.6 (4)
N7—Co2—N3—C21	29.0 (3)	Co1—N1—C3—C2	−3.0 (5)
O2—Co2—N3—C21	125.6 (3)	C1—C2—C3—N1	−49.4 (5)
Co1—Co2—N3—C21	163.7 (3)	C1—C2—C3—C4	126.7 (4)
O3—Co2—N3—C17	20.4 (3)	N1—C3—C4—C5	−0.2 (7)
O1—Co2—N3—C17	−36.2 (10)	C2—C3—C4—C5	−176.2 (4)
N10—Co2—N3—C17	110.4 (3)	C3—C4—C5—C6	1.8 (7)
N7—Co2—N3—C17	−155.9 (3)	C4—C5—C6—C7	−1.7 (7)
O2—Co2—N3—C17	−59.3 (3)	C3—N1—C7—C6	1.6 (6)
Co1—Co2—N3—C17	−21.2 (4)	Co1—N1—C7—C6	179.3 (3)
O3—Co1—N4—N5	−125.1 (3)	C5—C6—C7—N1	0.0 (7)
O2—Co1—N4—N5	−44.4 (3)	Co1—O2—C8—C9	32.7 (4)
O1—Co1—N4—N5	−72.5 (10)	Co2—O2—C8—C9	−72.9 (4)
N2—Co1—N4—N5	51.4 (3)	O2—C8—C9—C10	−68.6 (5)
N1—Co1—N4—N5	145.4 (3)	C14—N2—C10—C11	2.4 (6)
Co2—Co1—N4—N5	−83.7 (4)	Co1—N2—C10—C11	178.4 (3)
Co1—N4—N5—N6	172 (7)	C14—N2—C10—C9	−175.4 (3)
O3—Co2—N7—N8	52.6 (10)	Co1—N2—C10—C9	0.6 (5)
O1—Co2—N7—N8	36.3 (3)	C8—C9—C10—N2	50.3 (5)
N10—Co2—N7—N8	−59.3 (4)	C8—C9—C10—C11	−127.5 (4)
O2—Co2—N7—N8	114.9 (3)	N2—C10—C11—C12	−0.7 (6)
N3—Co2—N7—N8	−151.6 (3)	C9—C10—C11—C12	177.0 (4)
Co1—Co2—N7—N8	74.1 (4)	C10—C11—C12—C13	−1.0 (7)
Co2—N7—N8—N9	179 (100)	C11—C12—C13—C14	1.1 (7)
O3—Co2—N10—N11	151.3 (3)	C10—N2—C14—C13	−2.4 (6)
O1—Co2—N10—N11	−130.7 (3)	Co1—N2—C14—C13	−178.6 (3)
N7—Co2—N10—N11	−37.2 (3)	C12—C13—C14—N2	0.6 (6)
O2—Co2—N10—N11	172.6 (5)	Co1—O3—C15—C16	71.6 (4)
N3—Co2—N10—N11	54.3 (3)	Co2—O3—C15—C16	−36.6 (4)
Co1—Co2—N10—N11	−172.5 (3)	O3—C15—C16—C17	69.0 (4)
Co2—N10—N11—N12	−161 (4)	C21—N3—C17—C18	0.3 (6)
O3—Co1—O1—C1	−83.7 (3)	Co2—N3—C17—C18	−174.8 (3)
O2—Co1—O1—C1	−166.0 (3)	C21—N3—C17—C16	180.0 (4)
N4—Co1—O1—C1	−137.4 (8)	Co2—N3—C17—C16	4.9 (5)
N2—Co1—O1—C1	99.1 (3)	C15—C16—C17—N3	−51.8 (5)
N1—Co1—O1—C1	4.5 (3)	C15—C16—C17—C18	127.9 (4)
Co2—Co1—O1—C1	−124.7 (3)	N3—C17—C18—C19	0.8 (7)
O3—Co1—O1—Co2	41.02 (9)	C16—C17—C18—C19	−178.9 (4)
O2—Co1—O1—Co2	−41.27 (10)	C17—C18—C19—C20	−1.6 (8)
N4—Co1—O1—Co2	−12.7 (9)	C18—C19—C20—C21	1.3 (8)
N2—Co1—O1—Co2	−136.12 (11)	C17—N3—C21—C20	−0.7 (6)
N1—Co1—O1—Co2	129.19 (11)	Co2—N3—C21—C20	174.6 (4)
O3—Co2—O1—C1	81.9 (3)	C19—C20—C21—N3	−0.2 (8)

### Hydrogen-bond geometry (Å, °)

**Table:**

*D*—H···*A*	*D*—H	H···*A*	*D*···*A*	*D*—H···*A*
C6A—H6A1···N2B^i^	0.97	2.42	3.382 (3)	169
C8A—H8A2···N1A^ii^	0.96	2.57	3.384 (4)	142

Symmetry codes: (i) *x*−1, *y*, *z*; (ii) −*x*+1, −*y*, −*z*+2.
